# Effects of Testosterone Administration on Strategic Gambling in Poker Play

**DOI:** 10.1038/srep18096

**Published:** 2016-01-04

**Authors:** Jack van Honk, Geert-Jan Will, David Terburg, Werner Raub, Christoph Eisenegger, Vincent Buskens

**Affiliations:** 1 Department of Psychology, Utrecht University The Netherlands; 2 Institute of Infectious Disease and Molecular Medicine (IDM), University of Cape Town, South Africa; 3 Department of Psychiatry, University of Cape Town, South Africa; 4 Department of Psychology, Leiden University, The Netherlands; 5 Department of Sociology, Utrecht University, The Netherlands; 6 Department of Basic Psychological Research and Research Methods, University of Vienna, Austria

## Abstract

Testosterone has been associated with economically egoistic and materialistic behaviors, but -defensibly driven by reputable status seeking- also with economically fair, generous and cooperative behaviors. Problematically, social status and economic resources are inextricably intertwined in humans, thus testosterone’s primal motives are concealed. We critically addressed this issue by performing a placebo-controlled single-dose testosterone administration in young women, who played a game of bluff poker wherein concerns for status and resources collide. The profit-maximizing strategy in this game is to mislead the other players by bluffing randomly (independent of strength of the hand), thus also when holding very poor cards (cold bluffing). The profit-maximizing strategy also dictates the players in this poker game to never call the other players’ bluffs. For reputable-status seeking these materialistic strategies are disadvantageous; firstly, being caught cold bluffing damages one’s reputation by revealing deceptive intent, and secondly, not calling the other players’ bluffs signals submission in blindly tolerating deception. Here we show that testosterone administration in this game of bluff poker significantly reduces random bluffing, as well as cold bluffing, while significantly increasing calling. Our data suggest that testosterone in humans primarily motivates for reputable-status seeking, even when this elicits behaviors that are economically disadvantageous.

In a variety of species, the steroid hormone testosterone motivates individual animals to strive for social dominance^1^,^2^,^3^,^4^,^5^. Dominance behaviors triggered by testosterone are observed in male and female rodents, wolves, cattle, and primates including humans^6^,^7^,^8^,^9^,^10^. What these behaviors exactly are differs between these species; in pursuit of social dominance testosterone produces behaviors that range from flank-marking and physical aggression in rodents to threat signaling and staring endurance in primates, including humans^1^,^5^,^11^,^12^,^13^.

Social dominance ensures access to key resources, such as food, shelter and mating partners, and is thus highly adaptive for the individuals’ welfare and procreation. These huge benefits however do not come cheap, as “life at the top” is very stressful, and can lead to psychobiological adversities^14^,^15^. Alpha leaders in social species with a dominance hierarchy, whether male or female, not only guide and safeguard their social group, but also need to defend their status against competing group members^15^,^16^. Reputation matters, and social vigilance, strength, and resilience to stress and courage are required. Fortunately, testosterone not only motivates for social dominance, but also prepares to be socially dominant. The hormone increases social vigilance^17^,^18^, and strength^19^, and simultaneously reduces sensitivity to stress and fearfulness^7^,^20^,^21^,^22^.

In humans, however, status and resources are inextricably intertwined. High status, even given by birthright brings access to economic resources, and economic resources in turn convey, or buy, high social status. Consequently, in human societies, personal achievements and reputations matter less, as status can be ascribed or inherited^23^. Effects of testosterone depend on situation or environment^24^, thus in search for high status the hormone in our capitalistic society might have adaptively turned to materialism and greed. Indeed, proxies of current and prenatal testosterone correlate with the financial successes of trader’s on the stock market^25^,^26^, which is argued to indicate that the hormone adjusts behavior in humans in instrumental ways to maximize personal profits^27^. However, the adaptation went awry as greed induced by testosterone in the predominantly male banking world is assumed to have triggered, or accelerated the current economic crisis^27^,^28^,^29^. This financial debacle began when high testosterone bankers started strategically to gamble with public funds^29^,^30^,^31^. Greedy, amoral men caused the crisis and testosterone made them do it^29^,^30^,^31^,^32^. Testosterone is therefore considered an “immoral molecule”, and opposed to facilitating leadership eminence as it does in non-human animals, in humans the hormone is argued to be “the root to dysfunctional leadership”^33^. Less testosterone at the top is suggested to be necessary to prevent future economic crises^29^,^30^,^31^,^32^.

However, the notion that testosterone causes greed and immoral behavior is challenged by testosterone administration studies addressing human economic behavior. These studies show that, depending on situation and person factors^24^,^34^, testosterone can increase cooperation and fairness in economic interactions^35^,^36^. Testosterone administration furthermore has increased generosity in the Trust Game^37^, and the hormone decreased lying in a private game of dice^38^. These findings suggest that testosterone increases concerns for social reputation^35^,^36^,^37^,^38^, and that the hormone does not motivate for status seeking through either greedy or deceitful strategies^27^,^30^,^39^. However, concerns for status and resources remain hard to disentangle in humans as they are intrinsically intertwined^37^. Therefore, we investigated the effects of testosterone administration on behavior in a paradigm wherein concerns for status and resources uniquely collide: a two-person poker game developed by Von Neumann and Morgenstern^40^. We temporarily elevated the levels of testosterone in 20 young adult females with a validated sublingual 0.5-mg single-dose testosterone administration technique^20^,^41^,^42^,^43^, in a crossover, double-blind, placebo-controlled, within-subjects design^17^,^36^. Von Neumann and Morgenstern’s paradigm^40^ is a simplified poker game, streamlined to allow for experimental control. The essentials of poker, however, are preserved, that is, betting into a central pot, a hierarchy in hands, bluffing, folding, and calling. Importantly, a Nash equilibrium can be calculated, based on which clear predictions can be made in terms of behavioral strategies that maximize profits. Crucially, however, this strategy is in all its aspects disadvantageous for reputable status seeking. That is, the profit-maximizing strategy in this game is to mislead the other players by bluffing randomly (i.e. independent of strength of the hand), thus also when holding very poor cards (i.e. cold bluffing). The profit-maximizing strategy also dictates the players in this poker game to never call the other players’ bluffs. For reputable status seeking these materialistic strategies are disadvantageous; firstly, being caught cold bluffing damages one’s reputation by revealing deceptive intent, and secondly, not calling the other players’ bluffs signals submission in blindly tolerating deception.

Real-life poker begins by dealing out five cards, a so-called hand, out of a deck to each player. These hands are quantified in the game-theoretical framework by Von Neumann and Morgenstern^40^ as a random number within a continuous distribution between 0 and 1. Subsequently, both players simultaneously make a bet, either a “high” bet, ***(a)*** or a “low” bet ***(b).*** The most profitable strategy for placing a bet depends on the strength of the hand. For a sufficiently strong hand 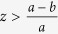 (>0.66) the player should always bid high. For weaker hands the player should bid irregularly high and low (with specified probabilities) to create uncertainty, and consequently protect the player against deviations from the equilibrium strategy of the opponent. In particular, the player should bet mostly ‘low’ on weaker hands, that is, with the probability 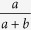. However, according to the equilibrium strategy players should place irregularly randomly distributed bluffs, that is, bet high with the probability 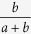. In our version of the game, high bets were 30 points (a = 30) and low bets were 10 points (b = 10). Thus, *bluffing* in this game is defined as placing a high bet with a hand <⅔ (that is,0 <0.67) and according to the Nash equilibrium the players should do this in 25% of the cases, independent of the actual strength of hands smaller than 0.67.

When players placed equal sized bets, hands are compared and the player with the higher hand wins the pot. However, when the bets are not equal the player with the lowest bet needs to decide if he/she wants to fold or to call. *Folding* similar to other poker games is that the player withdraws and that hands are not compared. Thus when folding the player pays the amount of his/her last bet, because this bet is lower than their opponent’s last bet. However, there is also an option for *calling.* The player can “call the bluff” of the opponent by placing a bet that matches the opponent’s bet. Hereby the bets are matched, and the players’ hands need to be compared (see Fig. 1).

In sum, the equilibrium strategy dictates players to bluff randomly in order to mislead the other players, with the probability of bluffing being independent of hand. This also includes bluffing with a very poor hand, i.e. cold bluffing. In addition, in the Nash equilibrium players should never call, as calling ends up to be costly in the long run on the basis of the game’s mathematics (see Supplementary Information). In stark contrast, however, playing according to the Nash equilibrium is disadvantageous for individuals seeking status on the basis of good reputation. Firstly, when an individual repeatedly bluffs with very weak hands (i.e., the cold bluffing approach), he/she runs the risk of being caught cheating in a weak position that damages reputation and results in a loss of status. Secondly, when the individual never calls the other players’ bluffs, he/she lacks control and consents to imminent deception, which signals submission. Calling on the other hand ultimately is economically costly. At present, we investigate the effects of testosterone administration on bluffing and calling in Von Neumann and Morgenstern’s Poker Game^40^, under the following premises: If testosterone administration compared to placebo results in an increase in random bluffing with more frequent cold bluffs and less calling, the hormone induces profit maximization. However, if after testosterone administration bluffing becomes more dependent on the strength of hand, with fewer cold bluffs and more calling, the hormone induces reputable status seeking. On the basis of our previous findings in testosterone administration studies, we also investigate whether a proxy of prenatal testosterone, 2D:4D digit ratio, modulates effects of testosterone on behavior^36^,^44^. Furthermore, we investigate relations between bluffing and calling behaviors in the Poker Game and self-reported traits of social dominance. Finally, we measure salivary testosterone levels, beliefs concerning condition and beliefs concerning effects of testosterone on behavior, to see whether these variables in any way mediate in the results (see Supplementary Information).

## Results

### Testosterone, Bluffing and Calling

**Figure 2:**
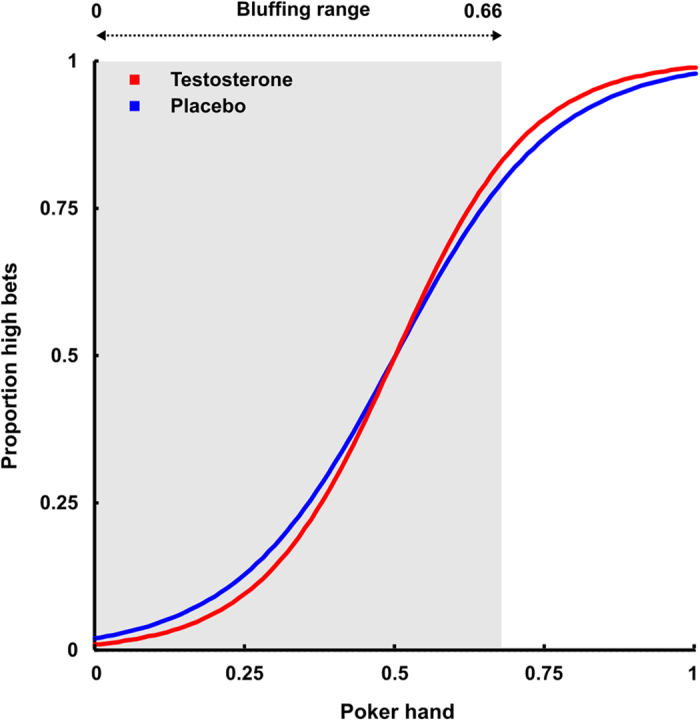
Proportion of high bets with regression lines for placebo and testosterone representing their interaction with hand in the multiple logistic regression model. Participants’ bluffing behavior depends on their poker hand, but this dependence is significantly stronger in the testosterone condition.

We first tested whether subjects performed according to the Nash equilibrium. Subjects bid high for hands with a value >⅔ in 92.9% of the cases after placebo, and in 94.4% of the cases after testosterone administration. Thus, subjects approached the theoretical optimum of 100% high bids in both conditions. Subjects also approached the optimal bluffing probability of 25%. They bid high for hands <⅔ in 25.9% of plays after placebo administration and in 25.3% of the cases after testosterone administration. The Nash equilibrium prescribes to bluff irregularly with this probability of 25% across the entire bluffing range, thus irrespective of the value of the hand, in order to be optimally unpredictable. Fixed effects logistic regression analyses (see Supplementary Information) showed that subjects’ bluffing performance was not optimally unpredictable, but dependent on hand; after placebo (ß = 7.69, SE = 0.42, Wald = 18.27, *p* < 0.001) and after testosterone administration (ß = 9.50, SE = 0.48, Wald = 19.62, *p* < 0.001). However, as the different ß values suggest, the crucial analyses of testosterone vs. placebo effects showed a significant difference in hand-dependent bluffing (hand x testosterone interaction: ß = 1.34, SE = 0.59, Wald = 2.26, *p* < 0.024). After testosterone administration, compared to placebo, bluffing depended more on hand, and therefore deviates more from the profit-maximizing strategy (see Fig. 2).

**Figure 3:**
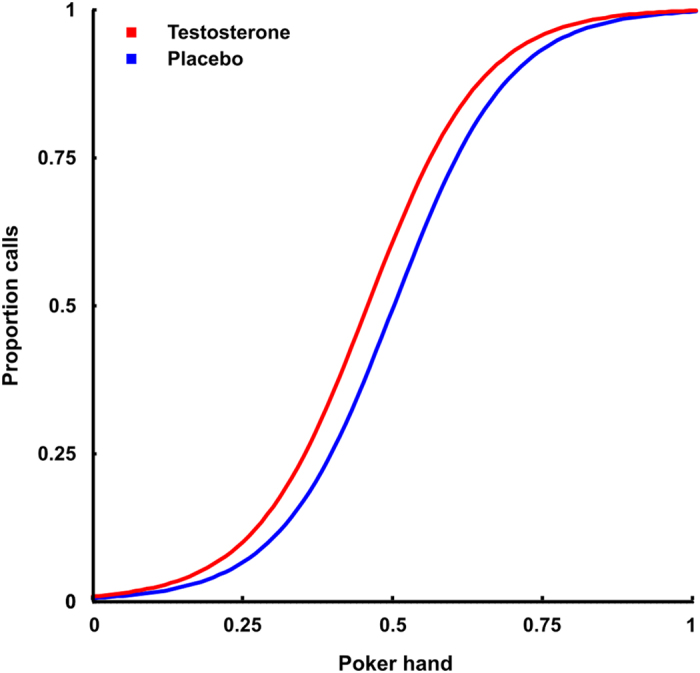
Proportion of calls with logistic regression lines for placebo and testosterone representing their main effect in the multiple logistic regression model. Testosterone increases calling.

To further qualify this interaction effect, the bluffing range was split in three equal-sized parts (low: 0.00–0.22; middle: 0.23–0.44; high: 0.45–0.66). This also allows insights in cold bluffing, that is, bluffing in a range wherein the option to win basically depends on convincing the opponent to fold, which arguably is the low range 0.00–0.22. Importantly, this a priori equal sized split and the cold bluffing range was objectified by a Bai-Perron multiple break point analysis (see Supplemental Information Statistical analyses and Figure S1). Results show that testosterone decreases bluffing significantly in the cold bluffing range (ß = −0.53, SE = 0.23, Wald = −2.28, *p* = .022). Furthermore, there were no effects of testosterone administration in the middle bluffing range (ß = 0.13, SE = 0.18, Wald = 0.71, *NS*) and the high bluffing range (ß = 0.19, SE = 0.13, Wald = 1.45, *NS*; see Fig. 3).

In sum, after testosterone administration participants, bluffing becomes more exploitable by the opponent, as bluffing is more strongly dependent on hand value and consistent with that, cold bluffing decreases. Thus, testosterone administration induces players to revert to a less profitable strategy.

**Figure 4:**
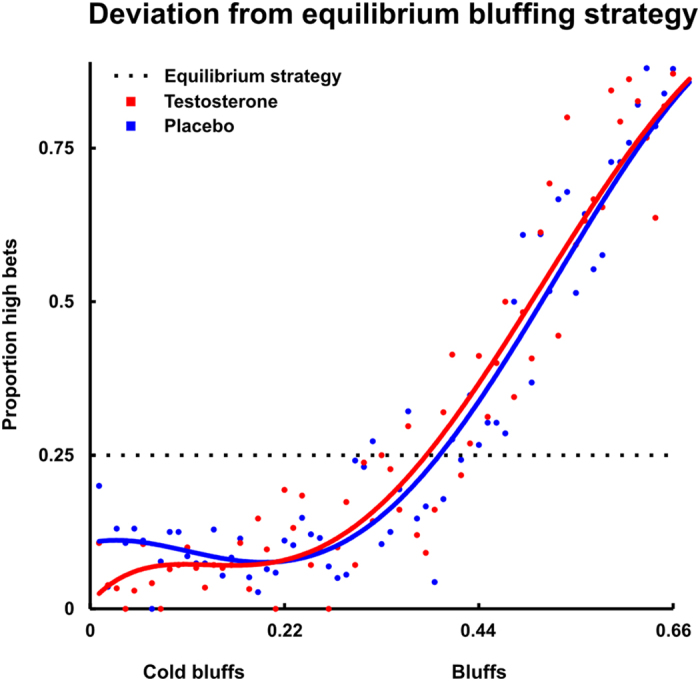
Visualisation of the influence of testosterone on bluffing behavior relative to the equilibrium strategy. Testosterone specifically reduces bluffing with weak hands, i.e. ‘cold bluffing’. Polynomial fits are based on the proportion of high bets with increment-size 0.01 over the full range of poker hands (0–1).

Next, we used fixed effects logistic regression analysis of the effects of testosterone on calling an opponent’s higher bet, which revealed an increase over the whole range after testosterone administration compared to placebo (main effect testosterone: ß = 0.59, SE = 0.27, Wald = 2.20, *p* = 0.028; **see** Fig. 4). Thus, after testosterone administration subjects call significantly more, which again is a less profitable strategy.

### Dominance Traits, Bluffing and Calling

To investigate how bluffing and calling in the Poker Game relate to traits of social dominance we administrated the Behavioral Activation Scales (BAS)^45^. On basis of earlier research we merged BAS reward and drive subscales into a measure of BAS-dominance, which, akin to the effects of testosterone administration, has been associated with implicit dominance behaviors measured by infrared eye-tracking^13^,^46^ (see Supplementary Information).

Random effects logistic regression analyses showed that there was a negative main effect of BAS-dominance on bluffing at baseline in the placebo condition (ß = −0.42, SE = 0.15, Wald = −2.85, *p* = 0.004). We next investigated whether this effect was most pronounced in the lower end of the bluffing range by again testing its 3 ranges. BAS- dominance significantly predicted less cold bluffing (ß = −0.88, SE = 0.38, Wald = −2.29, *p* = 0.022), and less bluffing in the middle range: (ß = −0.54, SE = 0.19, Wald = −2.78, *p* = 0.005) but had no effect on bluffing in the high range (ß = −0.12, SE = 0.27, Wald = −0.43, *NS).* In sum, we show that both testosterone administration and self-reported status seeking tendencies predict less cold bluffing, which substantiates our notion that cold bluffing is a disadvantageous strategy for those seeking high status.

Random effects logistic regression analyses showed that there were no effects of the BAS dominance scale on calling, at baseline, that is, in the placebo condition (ß = −0.25, SE = 0.29, Wald = −0.85, *NS*).

### Baseline Salivary Testosterone Levels and 2D:4D digit ratio

Subsequently, we investigated possible effects of testosterone administration on bluffing and calling, and its interaction with salivary testosterone levels and 2D:4D digit ratio. Previous studies have shown that effects of testosterone administration on social behaviour can vary strongly with the right hand’s digit ratio, a proxy of prenatal sex hormone priming^36^,^44^. Firstly, testosterone levels measured from saliva before administration did not differ between the testosterone and placebo administration condition, (t(18) = −1.52, *NS*). Furthermore, in the placebo condition salivary testosterone level did not predict bluffing (ß = 0.09, SE = 0.33, Wald = 0.26, *NS*) nor did it predict calling (ß = −0.12, SE = 0.67, Wald = −0.19, *NS*). Likewise in the placebo condition, 2D:4D digit ratio also did not predict bluffing (*p* = 0.47) nor did it predict calling (*p* = 0.24). Finally, salivary testosterone levels and 2D:4D digit ratio did not significantly interact with the effects of testosterone administration on bluffing and calling (all *p*s > 0.30).

Finally, we investigated if subjects were aware of condition (testosterone or placebo), and whether being aware of condition modulated the effects of testosterone administration on bluffing and calling. Subjects guessed at chance level (50% correct), which suggests no awareness. Moreover, even if they were correct in their believes about the condition they were in, this factor did not mediate in any way the effects of testosterone on bluffing and calling (see Supplementary Information).

## Discussion

In sum, our data show that after testosterone administration, compared to placebo, subjects bluff less randomly; their bluffing is more dependent on the strength of their hand. Bluffing under the influence of testosterone becomes thus more predictable and hence easier to foresee by the opponent. This is especially caused by subjects bluffing significantly less with very weak cards after testosterone administration; in the cold bluffing range. Both the reduction in random, as well as cold bluffing signifies a greater deviation from the game’s profit-maximizing strategy^40^. Bluffing should be unpredictable and occur over the whole range, and thus also with a very weak hand. A key reason for bluffing, according to Von Neumann and Morgenstern, is the aspiration to cheat by giving false impressions of strength in real weakness^40^. Note that in non-human animals, cheating behaviours are not prevalent in the dominant, but rather in the lower rank individuals. Alpha male or females generally have privileged access to partners and food resources, and do not require cheating. Their dominance typically is based upon real strength, which is challenged continuously. Subordinates on the other hand need deceptive strategies to get access to partners and food resources, and may receive punishment from the group leader when deception is detected^47^,^48^.

If testosterone in humans encourages dominance behaviour in ways corresponding to other animals, the hormone should not increase cheating behaviour, but instead improve reputation building and cheater detection. Indeed, there is evidence in humans that dishonest, treacherous behaviour is associated with low testosterone levels^49^. Additionally, as noted earlier, administration of testosterone has shown to induce reputable and honest behaviours in human males and females^11^,^12^,^13^,^42^. Reputation is slowly earned and quickly lost, and cheating may seriously compromise one’s reputation and one’s chances of benefitting from future cooperation^50^. Increases in prosocial behaviour observed in testosterone administration studies in humans may therefore serve reputation building, and mutually beneficial cooperation^25^,^26^,^51^.

We also show that testosterone administration caused subjects to call the bluffs of the opponents more in the poker game, and these data provide powerful corroborating evidence for testosterone’s proposed role in reputable-status seeking in humans^5^,^35^,^52^. The frequency of calling increased after testosterone administration in this poker game, wherein the profit-maximizing strategy is to never call^40^. Note that this profit-maximizing strategy, to always fold and never call the other players’ bluff, clearly is a submissive policy, as the player passively allows the other player to deceive and make profit through these cheats. Our prior research has repeatedly demonstrated that humans with exogenously increased, or endogenously high testosterone levels automatically and robustly avoid the display of submission signals^4^,^5^. The increased frequency of calling after testosterone administration in our Poker Game might therefore point at costly signalling strategies^53^,^54^. This includes the use of cost-ineffective behaviours to reliably signal dominance qualities^55^,^56^. Calling in the Von Neumann and Morgenstern poker paradigm might be such a costly dominance signal because it transmits information concerning the dominance qualities of an individual, in terms of controlling power, and determination for cheater detection^53^.

Taken together, after testosterone administration compared to placebo our subjects deviate significantly more from all profit-maximizing strategies in the Poker Game. The finding that testosterone administration induces more predictable and thus poor bluffing strategies, and less cold bluffing in particular, may run against typical beliefs of the effects of this hormone on behaviour. Indeed, debriefing showed that participants in the present study generally expected that testosterone would increase bluffing (see Supplementary Information). Furthermore, although testosterone has abundantly been associated with fearlessness and risk taking^20^,^42^,^43^, our bluffing data suggest that when social reputation is at stake, testosterone might actually induce a risk averse strategy. In other words, being caught cold bluffing, thus being exposed in a weak position “with the hand in the cookie jar” damages one’s reputation. This is a risk subjects with increased testosterone may not want to take. Our findings with self-reported traits of social dominance strongly support this notion, as we show that high trait-dominance^45^,^46^ significantly predicts reductions in cold bluffing. High trait dominance and high testosterone levels both predict economic risk taking in humans^45^,^57^, but to risk one´s social reputation is another matter as loss of dominance status is at stake.

In sum, the bluffing and calling strategies we show after testosterone administration are in all cases ultimately costly, and defensibly serve reputable-status seeking. These findings in humans translate to the effects of testosterone on dominance behaviours in many other species in whom social aptitude, costly signalling, cheater detection, fighting ability, and abilities to guide and protect the social group are essential in gaining and maintaining social status^15^,^16^. At the crossroad of status and resources, testosterone does not seem to induce materialistic, greedy behaviours in humans, but instead status-seeking behaviour based on reputation concerns.

## Methods

### Participants

Twenty female volunteers (age: 19–26 years; M = 21.1; SD = 1.9) participated in this double-blind, crossover, within-subject study. They received a single dose of 0.5 mg sublingual testosterone in one session and a single dose of placebo in the other session, with a 7-day latency between sessions. Subjects had no (history of) psychiatric disorders or neurological or endocrine abnormalities. They furthermore did not smoke, and used no medication other than contraceptive agents. We exclusively recruited women because the critical parameters (quantity and time course) for inducing neurophysiological effects after sublingual administration of testosterone are known in women but not in men (see Supplementary Information). We controlled for influences of hormonal change related to menstrual cycle by including only women who used single-phase contraceptives, and testing them during the 3-week period they were on these contraceptives and not during menstruation (see Supplementary Information). The medical ethics committee of the University Medical Centre Utrecht, The Netherlands, approved the protocol of this study. The study was carried out in accordance with these approved guidelines, and informed consent was obtained from all subjects.

### Generalizability of Effects to Males

As noted above, the parameters (quantity and time course) for inducing neuro-physiological effects are known in women but not men. As a result, in humans there is an abundance of research testing the effects of single administrations of testosterone on social and emotional behavior in women, but very little research in men^24^. However, on the basis of both animal and human data with both endogenous and exogenous testosterone, effects on dominance behaviors and fear behavior are sex independent^6^,^7^,^8^,^9^,^10^,^20^,^21^,^22^,^43^,^44^. We have repeatedly shown that single administration of testosterone in young women result in more male-typical social and affective behavior (i.e. reductions in both cognitive empathic abilities and stress responsivity)^18^,^21^. As noted, recent studies on social-economic decision-making have shown that single dose administration of testosterone in both females and males produce results that can best be explained in terms of increases in status seeking behavior^35^,^36^,^37^,^38^. Finally, pharmacological neuroimaging studies have shown that single administrations of testosterone produce similar effects on the neural circuits of threat/dominance in females and males^58^,^59^. Summarizing, the relation between testosterone and human emotional and social-emotional behavior in the domains of dominance, fear and social decision-making (which cover the behaviors addressed here) is very similar in males and females. We therefore expect that the current findings will generalize to males.

### Zero-sum two-person poker game

A computerized zero-sum two-person poker game was built based upon the framework of Von Neumann and Morgenstern^40^. In this game each player is endowed with 4500 points in each testing session, with 30 points = € 0.10. In each round of the game both players are dealt a random hand X ∈ [0,1] in 5 decimals, where X has a uniform distribution over the interval [0,1]. Subsequently, both players are asked to simultaneously make a discrete bet, with 2 options only i.e. high (30 points) or low (10 points). When the bets match, hands are disclosed and compared, and the player with the higher hand wins the pot. When the bets do not match, there was a second phase in the game in which the player with the low bet can *call* (raise the bet from 10 to 30 points), or *fold*. If the player with the low bet calls, hands were compared and the player with the highest hand wins the pot. If the player with the low bet folds, hands are not compared and the player with the higher bet wins the pot.

### Statistical Analyses

Our main statistical analyses of the effects of testosterone on bluffing and calling are based on fixed-effects multiple logistic regressions. Interaction terms were mean centred (variable – mean variable) to reduce multicollinearity and to increase the interpretability of the interaction. We used a random effects logistic model to analyse individual differences at baseline in bluffing and calling in relation to salivary testosterone levels, 2D:4D digit ratio and BAS dominance scores.

## Additional Information

**How to cite this article**: van Honk, J. *et al.* Effects of Testosterone Administration on Strategic Gambling in Poker Play. *Sci. Rep.* **6**, 18096; doi: 10.1038/srep18096 (2016).

## Supplementary Material

Supplementary Information

## Figures and Tables

**Figure 1:**
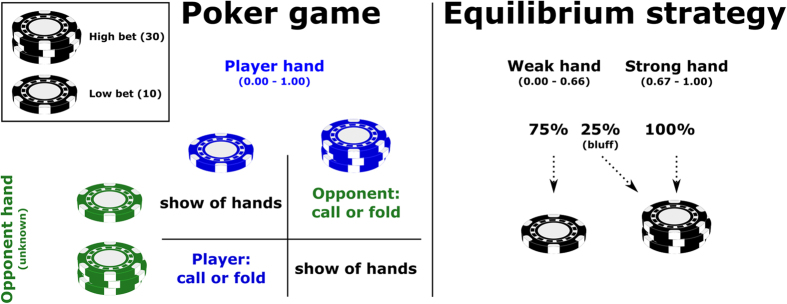
Mechanistics of the Von Neumann and Morgenstern poker game and its equilibrium strategy.
