# Erratum: Effects of Testosterone Administration on Strategic Gambling in Poker Play

**DOI:** 10.1038/srep21745

**Published:** 2016-02-25

**Authors:** Jack van Honk, Geert-Jan Will, David Terburg, Werner Raub, Christoph Eisenegger, Vincent Buskens

Scientific Reports 6: Article number: 1809610.1038/srep18096; published online: 01042016; updated: 02252016.

The HTML version of this Article contains an error in the order of the Figures and their legends. Figures 3 and 4 were published as Figs 4 and 3 respectively.

The PDF version of this Article contains an error in the order of the Figure legends. Figure legends 3 and 4 were published as Figure legends 4 and 3 respectively. The Figures are correct.

The correct Figs 3 and 4 and their accompanying legends appear below as Figs 1 and 2 respectively.

## Figures and Tables

**Figure 1:**
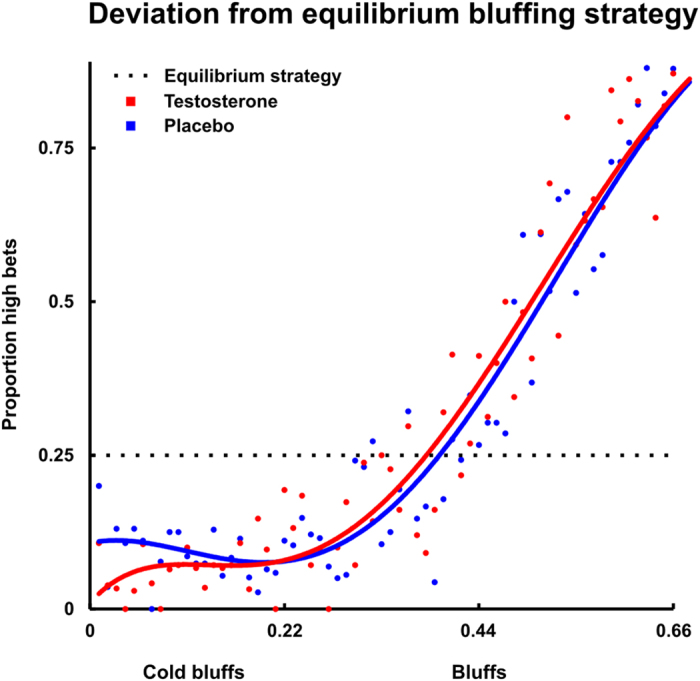
Visualisation of the influence of testosterone on bluffing behavior relative to the equilibrium strategy. Testosterone specifically reduces bluffing with weak hands, i.e. ‘cold bluffing’. Polynomial fits are based on the proportion of high bets with increment-size 0.01 over the full range of poker hands (0–1).

**Figure 2:**
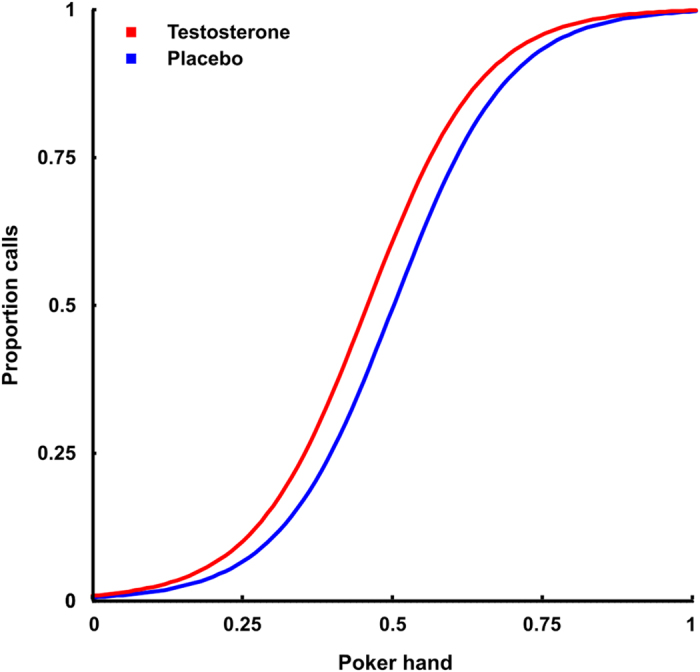
Proportion of calls with logistic regression lines for placebo and testosterone representing their main effect in the multiple logistic regression model. Testosterone increases calling.

