# Supplementary material for: Effects of Testosterone Administration on Strategic Gambling in Poker Play
Source: Sci Rep. 2016 Jan 4;6:18096. doi: 10.1038/srep18096 (PMC4698749; doi:10.1038/srep18096)
Supplement: Supplementary Information [file srep18096-s1.pdf]

SUPPLEMENTARY MATERIALS

Effects of Testosterone Administration on Strategic Gambling in Poker Play

Jack van Honk, Geert-Jan Will, David Terburg, Werner Raub,
Christoph Eisenegger, Vincent Buskens.

Participants

Twenty female volunteers (age range, 18-30) participated in this double-blind, crossover and within-subject study. All participants gave informed consent and the protocol of the study has been approved by the medical ethics committee (METC) of the University Medical Centre Utrecht, The Netherlands. Participants received a single dose of 0.5 mg sublingual testosterone in one session and placebo in the other session, with a 7-day latency between sessions. They received payment consisting of €10 as a fixed fee plus earnings depending on their performance in the poker game. Subjects had no (history of) psychiatric disorders or neurological or endocrine abnormalities, did not smoke, and used no medication other than contraceptive agents. We controlled for influences of hormonal change related to menstrual cycle by including only women who used single-phase contraceptives (with 0.15 mg levonorgestrel and 0.03 mg ethinylestradiol), and tested them during the 3-week period they were using these contraceptives, but not during menstruation [1]. We exclusively recruited women because the parameters (quantity and time course) for inducing neurophysiological effects after a single sublingual administration of 0.5mg of testosterone are known in women but not in men [2,3]. These parameters, that is, 0.5 mg testosterone with and measurements 4 hours after administration were established using vaginal pulse amplitude (VPA), a uniquely non-habitual, centrally driven response evoked by erotic material[2]. To date, no method is available to assess the time course of effects of testosterone in human males. Crucially, behavioral effects after a 4-h delay has been successfully established in more than 25 studies, addressing sexual, social, and emotional behaviors in young women (e.g.[4-10]).

Zero-sum two-person poker game

We created a computerized version of a zero-sum (i.e. one player's loss is the other player's gain) two-person poker game based on the seminal work of Von Neumann and Morgenstern[11]. Each player was endowed with 4500 points in each testing session (with 30 points = € 0.10). At the start of each round of the game players are dealt a random hand X [0,1] in 5 decimals, with X being uniformly distributed over the interval [0,1]. The value of their own hand gives no information about the value of the opponents' hand. Players are subsequently asked to simultaneously make a discrete bet: high (30 points) or low (10 points). If players placed the same bets, their hands were disclosed and compared, the player with the highest hand wins the pot (consisting of the shared points). If players place different bets, that is, one bets high and the other low, the player with the lower bet can then either *call* (match the opponent's bet by raising to 30 points) or *fold*. If a player calls both hands are compared and the player with the highest hand wins the pot. If a player folds, hands are *not* compared and the player with the highest bet wins the pot. Thus, this provides for the opportunity to bluff. A bluff is defined as a high bet (a) for hands weaker than , according to the rationale in [11]. In our study bets were a = 30 and b = 10, thus a bluff was betting 30 points for hands lower than . A round of poker ends if either both players bet the same amount of points or if one of the players folded. Each participant played 150 rounds of poker on each testing day, of which 50 rounds were played against each opponent. Final earnings were not disclosed until the end of the second testing day to make sure that knowledge about the profits on the first day, would not influence the participants' strategy on the second testing day. Participants played the game in a cubicle on a computer and were connected with the other participants' using Z-tree 3.0 software[12].

Social dominance

We used the combined subscales Reward responsiveness (BASR) and Drive (BASD) of the Behavioral Activation Scale (BAS), which is a validated measure of social dominance. These BAS scales in measuring approach motivation and reward sensitivity[13], tap into dominance as established previously using social-affective reaction time and infrared eye-tracking paradigms[14-17]. Neuroeconomic research has shown that BASD and BASR predicts higher offers in the Ultimatum Game[18], which are argued to point at increased concerns for social status[19].

Mood

The shortened version of the Profile of Mood States [20] was used to index possible effects of testosterone on anger, tension-anxiety, fatigue, vigor, and depression. There were no significant differences on any of these subscales between the testosterone and placebo conditions (all p's > .05), in line with our previous observations[3]. This precludes any alternative explanations of our effects in terms of secondary mood-generated changes[21]

2D: 4D digit ratio

Individual digit ratios were measured from an image scan of the right-hand by taking the length of the index and ring finger from the ventral proximal crease to the tip of the finger using an Adobe Photoshop measurement tool[22]. An experienced rater who was blind to the goals of the experiment, measured 2D: 4D twice, with a latency of several weeks. These two measurements were highly correlated ($r = .99$, $p < .0001$). The mean value of the two measurements was used for analysis (see Statistical analyses).

Salivary Testosterone

Testosterone in saliva was measured after diethylether extraction using a competitive radioimmunoassay employing a polyclonal antitestosterone antibody (AZG 3290; a gift from J. J. Pratt, Groningen, The Netherlands). [1,2,6,7-3H]-Testosterone (TRK402; Amersham Nederland BV) was used as a tracer following chromatographic verification of its purity. The lower limit of detection was 10 pmol/L, and interassay variation was 16.1, 11.5, and 5.1% at 21, 100, and 230 pmol/L, respectively (n = 4, 5, 5). Samples of one subject were not available, and sample of a second subject was contaminated on the basis of abnormal range. Main analyses concern samples of 18 subjects in placebo condition.

Statistical analyses

For bluffing behaviour, we restrict our analysis to initial bets for hands < ⅔ according to theoretical framework of the game[11]. We examined the impact of testosterone [binary indicator for testosterone (1) or placebo (0)], hand (continuous variable ranging from 0 to 1), and interaction term (testosterone x (hand – mean hand) on the probability of betting "high" (= 1, betting "low" = 0) as the dependent variable.. To qualify the testosterone x hand interaction observed for bluffing behaviour, the bluffing range was split into three equal-sized parts (0 - .22; .23 - .44; .45 - .66) on the basis of a priori hypotheses concerning cold bluffing. Crucially however, we objectively defined the hand ranges wherein the overall (i.e. placebo

and testosterone conditions combined) relationship between betting behaviour and hand changed significantly. We applied a moving average to the logistic betting distribution (first bets (B) only with betting "high" B=1 and betting "low" B=0), which was subsequently transformed to its linear equivalent by applying a natural logarithm to the odds-ratio of high bets (lnOR with lnOR(B)=ln(B/(1-B))). The moving average was also applied to the poker-hand data and its window was set to include all observations between hand(H)=H and hand(H)=H+0.05, which ensured elimination of the occurrence of undefined lnOR values (i.e. lnOR(0)=ln(0/1) and lnOR(1)=ln(1/0)). Next, we used a Bai-Perron multiple break point test[23] to identify significant ($p < .05$) changes in the coefficients of a linear regression of lnOR(B) on hand(H). Two break points were identified that indicated a significant change in betting behaviour on Hand=0.20 and Hand=0.36 (see **Figure S1** for the associated regression lines and coefficients).

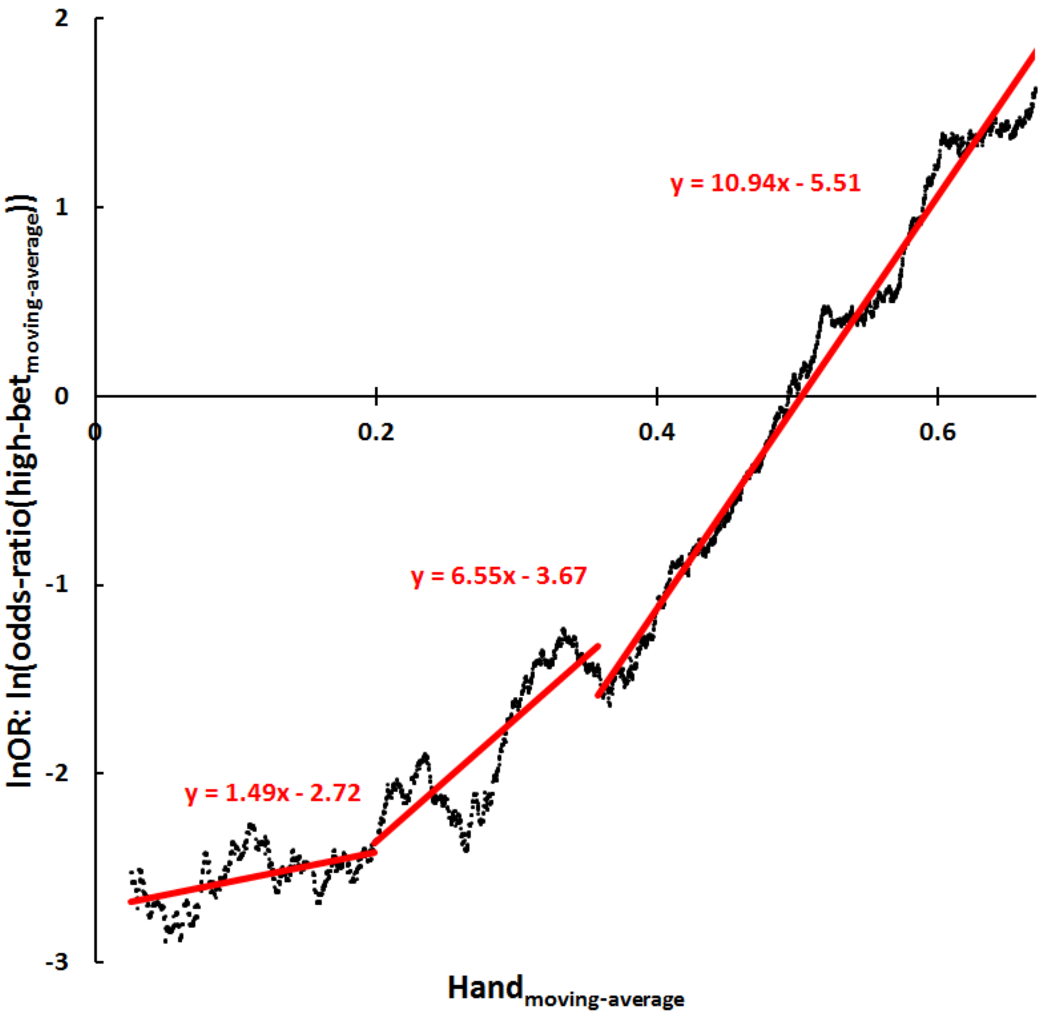

Figure S1 Bai-Perron multiple break point analysis on the relation between betting behaviour and poker-hand over the whole data set (i.e. placebo and testosterone sessions combined) plotted for the

full bluffing range. Significant changes in bluffing behaviour were identified at Hand=0.20 and Hand=0.36.

The intervals on the basis of these break points largely agree with our a priori three-way split. Moreover, all main results are confirmed using these break points: For the bluffing behaviours in (the cold bluffing) range 0 – 0.20, there was a main effect of testosterone (β = -.62, *SE* = 0.25, *Wald* = -2.47, *p* = .014) and a main effect of BAS-dominance (β = -.69, *SE* = 0.27, *Wald* = -2.52, *p* = .012). For the bluffing behaviours in range 0.20 – 0.36, there was no effect of testosterone (β = .16, *SE* = 0.23, *Wald* = 0.69, *p* = .489), but there was a main effect of BAS-dominance (β = -.61, *SE* = 0.18, *Wald* = -3.40, *p* = .001). For the bluffing behaviours in range 0.36 – 0.67, there was neither an effect of testosterone (β = .20, *SE* = 0.12, *Wald* = 1.72, *p* = .086), nor an effect of BAS-dominance (β = -.20, *SE* = 0.25, *Wald* = -0.79, *p* = .427)
For the calling behaviours, we examined the impact of testosterone (1) or placebo (0)], hand (continuous variable ranging from 0 to 1), and an interaction term (testosterone x (hand – mean hand) on the probability of betting "calling" (= 1) as the dependent variable (0 = "folding"). We analysed individual differences (i.e. salivary testosterone levels, 2D: 4D digit ratio and BAS-dominance scores) using *random effects* logistic models. In these models "betting" was the dependent variable (0: betting low; 1: betting high) and hand, BAS scores and hand x BAS scores as well as salivary T levels and hand x salivary T interaction terms were predictor variables. On the basis of our previous studies in which we report effects of testosterone on behaviour modulated by the right hand's second-to-fourth-digit ratio, a proxy of prenatal hormonal priming [8,24,25], we performed a full *random effects* logistic model with: dependent variable (0: betting low; 1: betting high) and predictors: hand, testosterone/placebo administration (1 or 0), digit ratio, two-way interaction terms (testosterone x (hand – mean hand); digit ratio x hand; digit ratio x testosterone administration) and a three-way interaction term (hand x T administration x digit ratio) as predictor variables.

Beliefs about the effects of testosterone on poker play

Most of the participants reported that they believed that testosterone administration: would: 1) Increase a desire to gamble (90% reported that testosterone would increase a desire to gamble; 10% had no idea); 2) Increase a willingness to take risks (95% increase; 5% no idea); 3) Increase bluffing 65% (35% no idea); 4) Increase calling (60% increase (40% no idea). Importantly, there was thus not one participant who believed that testosterone administration would decrease a desire to gamble, willingness to take risks, bluffing or calling.

Beliefs concerning condition

On the final day of the experiment participants were asked on what day they believed to have received testosterone, that is, 1st or 2nd day or whether they had no idea. 10 guessed correctly and 10 guessed wrong or had no idea. To test whether participants' correct belief of having received testosterone in any way interacted with our effects of testosterone on bluffing and calling, we ran the bluffing and calling analyses again with the binary "belief" indicator denoting whether the participant guessed correctly (1) or not (0) as covariate in random effects analyses. The reported bluffing and calling effects remained significant, and the covariate "belief" did not predict bluffing ($p = .225$) nor calling behavior ($p = .216$).

Beliefs about the effects of testosterone on gambling, bluffing and calling

The majority of participants reported that they believed that testosterone administration would: 1) increase a desire to gamble (90% reported that testosterone would increase a desire to gamble; 10% had no idea); 2) increase a willingness to take risks (95% increase; 5% no idea); 3) increase bluffing (65% more often place higher bets for hands < .50; 35% no idea); and 4) increase calling (60% increase; 40% no idea). Specifically, no participants reported that they expected testosterone administration to: 1) decrease a desire to gamble, 2) decrease a willingness to take risks, 3) decrease bluffing, or 4) decrease calling.

References

1 Zhang, D., Wang, L., Luo, Y. & Luo, Y. Individual Differences in Detecting Rapidly Presented Fearful Faces. *PloS one* **7**, e49517 (2012).

2 Tuiten, A. *et al.* Time course of effects of testosterone administration on sexual arousal in women. *Arch. Gen. Psychiatry* **57**, 149-153; discussion 155-146 (2000).

3 Bos, P. A., Panksepp, J., Bluthé, R. M. & van Honk, J. Acute effects of steroid hormones and neuropeptides on human social-emotional behavior: A review of single administration studies. *Front. Neuroendocrinol.* **33**, 17-35 (2012).

4 van der Made, F. *et al.* Childhood sexual abuse, selective attention for sexual cues and the effects of testosterone with or without vardenafil on physiological sexual arousal in women with sexual dysfunction: A pilot study. *Journal of Sexual Medicine* **6**, 429-439 (2009).

5 van Honk, J., Montoya, E. R., Bos, P. A., van Vugt, M. & Terburg, D. New evidence on testosterone and cooperation. *Nature* **485**, E4-5; discussion E5-6 (2012).

6 van Honk, J., Peper, J. S. & Schutter, D. J. Testosterone reduces unconscious fear but not consciously experienced anxiety: Implications for the disorders of fear and anxiety. *Biol. Psychiatry* **58**, 218-225 (2005).

7 van Honk, J. & Schutter, D. J. Testosterone reduces conscious detection of signals serving social correction: Implications for antisocial behavior. *Psychol. Sci.* **18**, 663-667 (2007).

8 van Honk, J. *et al.* Testosterone administration impairs cognitive empathy in women depending on second-to-fourth digit ratio. *Proc. Natl. Acad. Sci. U. S. A.* **108**, 3448-3452 (2011).

9 van Honk, J. *et al.* Testosterone shifts the balance between sensitivity for punishment and reward in healthy young women. *Psychoneuroendocrinology* **29**, 937-943 (2004).
10 Hermans, E. J. *et al.* Exogenous testosterone attenuates the integrated central stress response in healthy young women. *Psychoneuroendocrinology* **32**, 1052-1061 (2007).
11 Stoop, R. Neuromodulation by oxytocin and vasopressin. *Neuron* **76**, 142-159 (2012).
12 Fischbacher, U. Z-Tree: Zurich Toolbox for Ready-made Economic Experiments. *Experimental Economics* **10**, 171-178 (2007).
13 Carver, C. S., White, T.L. Behavioral inhibition, behavioral activation, and affective responses to impending reward and punishment: The BIS/BAS scales. *J. Pers. Soc. Psychol.* **67**, 319-333 (2004).
14 Putman, P., Hermans, E. & van Honk, J. Emotional Stroop performance for masked angry faces: It's BAS, not BIS. *Emotion* **4**, 305-311 (2004).
15 Terburg, D., Hooiveld, N., Aarts, H., Kenemans, J. L. & van Honk, J. Eye tracking unconscious face-to-face confrontations: Dominance motives prolong gaze to masked angry faces. *Psychol. Sci.* **22**, 314-319 (2011).
16 Terburg, D., Aarts, H. & van Honk, J. Testosterone affects gaze-aversion from angry faces outside of conscious awareness. *Psychol. Sci.* **23**, 459-463 (2012).
17 Terburg, D., van Honk, J. Approach–Avoidance versus Dominance–Submissiveness: A Multilevel Neural Framework on How Testosterone Promotes Social Status. *Emotion Review* **5**, 301-311 (2013).
18 Scheres, A. & Sanfey, A. G. Individual differences in decision making: Drive and reward responsiveness affect strategic bargaining in economic games. *Behavioral and Brain Functions* **2**, 35 (2006).
19 Eisenegger, C., Naef, M., Snozzi, R., Heinrichs, M. & Fehr, E. Prejudice and truth about the effect of testosterone on human bargaining behaviour. *Nature* **463**, 356-359 (2010).
20 Shacham, S. A shortened version of the Profile of Mood States. *J. Pers. Assess.* **47**, 305-306 (1983).
21 Kapur, S., Phillips, A. G. & Insel, T. R. Why has it taken so long for biological psychiatry to develop clinical tests and what to do about it[quest]. *Mol. Psychiatry* **17**, 1174-1179 (2012).
22 Breedlove, S. M. Minireview: Organizational hypothesis: Instances of the fingerpost. *Endocrinology* **151**, 4116-4122 (2010).
23 Bai, J. & Perron, P. Estimating and testing linear models with multiple structural changes. *Econometrica* **66**, 47-78 (1998).
24 van Honk, J., Montoya, E. R., Bos, P. A., van Vugt, M. & Terburg, D. New evidence on testosterone and cooperation. *Nature* **485**, E4-E5 (2012).
25 Montoya, E. R. *et al.* Testosterone administration modulates moral judgments depending on second-to-fourth digit ratio. *Psychoneuroendocrinology* **38**, 1362-1369 (2013).
